# Metastases From Nested Variant Urothelial Carcinoma of the Urinary Bladder in Pancreatic Allograft Mimicking Graft Rejection

**DOI:** 10.4021/jocmr798w

**Published:** 2012-03-23

**Authors:** Jue Wang, Geoffrey Talmon, Syed A. Jaffar Kazmi, Larry E. Siref, Michael C. Morris

**Affiliations:** a Department of Internal Medicine, Section of Oncology-Hematology, University of Nebraska Medical Center, Omaha, Nebraska 68198-7680, USA; b Department of Pathology and Microbiology, University of Nebraska Medical Center, Omaha, Nebraska 68198-3135, USA; c Urologic Surgery Section, Department of Surgery, University of Nebraska Medical Center, Omaha, NE 68198-2360, USA; d Transplant Surgery Division, Department of General Surgery, University of Nebraska Medical Center, Omaha, NE, USA

## Abstract

While not an uncommon tumor, urothelial carcinoma of the urinary bladder is rare in bladders draining pancreatic allografts. A case of urothelial carcinoma directly involving a pancreatic allograft with metastasis that occurred in a 49-year-old pancreas and kidney transplant recipient is described. Her initial clinical presentation and findings of CT scan of the abdomen suggested pancreatitis with features worrisome for rejection. A biopsy of her pancreatic allograft contained poorly differentiated carcinoma and cystoscopic biopsy disclosed an invasive high grade urothelial carcinoma arising in the background of extensive urothelial carcinoma in situ. Exploratory laparotomy revealed that the tumor invaded the right ovary and fallopian tube, cecum, and allograft with extensive retroperitoneal involvement. She underwent en bloc resection of distal ileum and cecum, resection of transplant pancreas, partial cystectomy, ileocolostomy anastomosis, and right salpingo-oophorectomy. Postoperatively, the patient was treated with four cycles of carboplatin and gemcitabine. She ultimately succumbed to her disease approximately 1 year after diagnosis. This case should alert physicians and radiologists to be aware of atypical presentation of urothelial carcinoma in bladder-drained pancreas grafts, the aggressiveness of such lesions, and the need for early biopsy to avoid diagnostic confusion with rejection.

**Keywords:**

Bladder cancer; Nested variant of urothelial carcinoma; Pancreas and kidney transplantation

## Introduction

Bladder malignancy in the pancreas and kidney transplant recipient is rare with only a limited number of reported cases [1, 2]. Although the incidence of bladder cancer in organ recipients appears to parallel that of the general population, the tumors are more likely to be high grade with an aggressive clinical course and poor response to chemotherapy. Among transplanted patients, the age of onset is younger (average 44 years) without the typical male predominance [3-5]. The duration of immunosuppression associated with the development of the urothelial malignancy varies from 2 years to >10 years [3].

Here, this report illustrates an unusual presentation of urothelial carcinoma of the urinary bladder in a 49-year-old kidney/pancreas transplant recipient which was initially thought clinically and radiologically to represent pancreatic graft rejection.

## Case Report

A 49 year-old Caucasian woman was transferred to our hospital with right flank and lower quadrant abdominal pain in March 2010. Her history was significant for type I diabetes mellitus, hypertension, hyperlipidemia, end-stage kidney disease. She was status-post two pancreatic transplants in 1991 and 2006, as well as and four renal transplants (last in February 2000). Seven months prior to this admission, she was diagnosed with a low grade noninvasive papillary urothelial carcinoma. A repeat cystoscopy two months before this admission was negative for any gross evidence of tumor recurrence.

**Figure 1:**
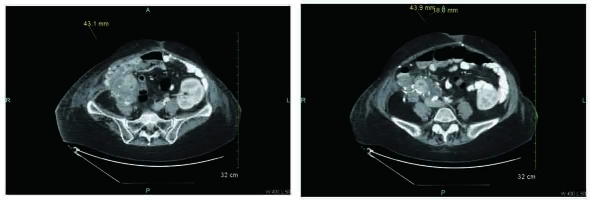
CT scan of abdomen reveals peripancreatic fluid collections with involvement of the cecal pole, findings suggestive for pancreatitis around the transplant. The pancreas transplant in the right lower quadrant is surrounded by inflammation and two fluid density areas. The inflammatory changes extend into the cecal pole area and involve this bowel loop. The inflammatory changes also extend towards the bowel, close to the urinary bladder. There is no fatty plane between the urinary bladder and the pancreas transplant with inflammation. The findings were initially concerning for pancreatitis with graft rejection.

On admission, a computed tomography (CT) scan of the abdomen revealed peripancreatic fluid collections with involvement of the cecal pole and urinary bladder. Findings were suggestive of pancreatitis and worrisome for rejection (Fig. 1). A biopsy of her pancreatic allograft revealed diffuse involvement by poorly differentiated carcinoma. A cystoscopy with biopsy revealed an invasive high grade urothelial carcinoma arising in the background of extensive urothelial carcinoma in situ. A positron emission tomography (PET scan) showed an abnormally increased signal in the region of the pancreas, and anterior abdominal wall. Exploratory laparotomy revealed extensive tumor invading the right ovary and tube, the cecum, and the transplant pancreas with extensive retroperitoneal involvement. Subsequently, she underwent en bloc resection of distal ileum and cecum, resection of transplanted pancreas, partial cystectomy, right salpingo-oophorectomy, and repair of ileocolostomy anastomosis.

**Figure 2:**
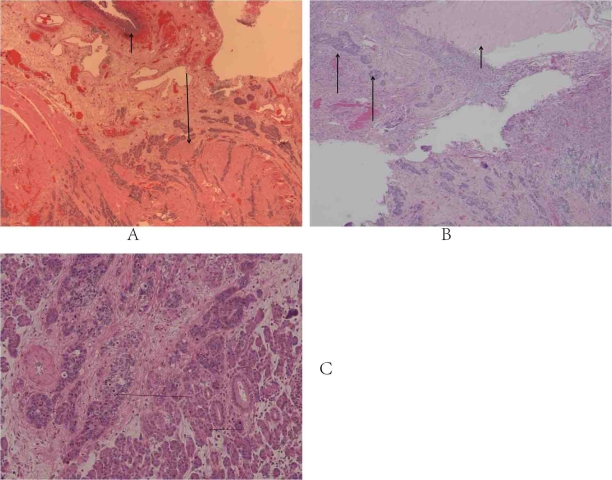
Photomicrograph of specimen from (A) cystectomy specimen. Short arrow points to adjacent normal urothelium, long arrow to nests of tumor cells invading muscularis propria. (B) Ovary. Ovary involved by carcinoma via direct extension. Long arrows: tumor in ovarian stroma. Short arrow: corpus albicans. (C) The transplant pancreas. The long arrow points to nests of invasive carcinoma and the short arrow highlights the pancreatic parenchyma. The high-grade urothelial carcinoma shows a predominant invasive nested variant of urothelial carcinoma (NVUC) pattern.

Pathologic examination of the resection specimen disclosed a 4.9 cm mass within bladder cuff near the allograft that directly invaded the right ovary, fallopian tube, cecum, and pancreas allograft (Fig. 2), as well as extensive retroperitoneal involvement. The tumor demonstrated a prominent nested growth pattern reminiscent of the nested variant of urothelial carcinoma (NVUC) with other areas showing features more typical of conventional invasive high grade urothelial carcinoma. The neoplastic cells were positive for pancytokeratin and OC125 (cytoplasmic) while negative for chromogranin, synaptophysin, CD56, CK7, CK20, CDX2, TTF1, ER, PR, p53, and BRST2. While not entirely specific, the staining pattern combined with the presence of adjacent urothelial carcinoma in situ was supportive of an urothelial origin. In addition, the lesions resected from her abdominal wall were positive for metastatic urothelial carcinoma.

Postoperatively, the patient was given with four cycles of gemcitabine and carboplatin, which completed in August 2010, with no measurable disease noted radiographically following therapy. In Feb 2011, the patient was admitted to jospital for worsening of abdominal pain. A CT scan revealed multiple intra-abdominal and peritoneal nodules consistent with metastatic disease. She went on hospice and died on March 2011.

## Discussion

Bladder cancer developing in organ transplant recipients remains a challenging disease to manage as it has been demonstrated that the clinical course seems worse than in the general population [1-4]. The immunosuppressed status of the transplant recipients renders the therapy and post-treatment surveillance very difficult [5]. With the increase of organ transplantation, urological cancer (including bladder cancer) may pose a critical problem affecting the survival of these patients.

NVUC was recently classified by the World Health Organization as an “uncommon aggressive tumor”, with few reported cases and a 70% mortality rate 4 to 40 months after diagnosis despite therapy [6]. The incidence of NVUC has been estimated to be 0.8% of all invasive bladder carcinoma [7] and fewer than 100 cases have been reported [8-11]. This variant exhibits aggressive clinical behavior with rapid spread along the lymphatics in the lamina propria of the urinary bladder and along lymphatic channels into the peritoneum [8].

While the degree of cytologic atypia noted in this case is not typically described in NVUC, this feature can be seen in these lesions and NVUC is associated with areas of conventional high grade urothelial carcinoma in the majority of instances [12]. In the present case, her pancreas is bladder drained and it is possible that the atypia noted may be related to the effect of exocrine pancreatic secretions. Indeed, clinical behavior and pattern of spread is compatible with NVUC and cases with nested features have a poor outcome [12].

To our knowledge, this is the first case of urothelial carcinoma demonstrating NVUC features reported in the transplant receipt, the tumor invaded the transplanted pancreas with extensive retroperitoneal involvement. This is a unique presentation that clinically mimicked pancreatitis and/or rejection. The rapid progression from a clinically nonapparent lesion widely invasive disease may be related to the patient’s immunosuppressive status, although as noted above lesions with nested growth patterns often demonstrate an aggressive phenotype.

The optimal modality of treatment is uncertain because NVUC is rare and no randomized studies specifically designed for this subtype of bladder tumor [7, 10]. Traditionally, the standard therapy for patients with locally advanced or metastatic urothelial carcinoma is chemotherapy using methotrexate, vinblastine, doxorubicin, and cisplatin (MVAC) [13]. The gemcitabine-cisplatin regimen has been shown to have equivalent overall response rates, with less toxicity (range 41% to 57%), with a complete response in 15% to 22%, and a median survival of 12.5 to 14.3 months [14]. Although this subtype of urothelial carcinoma is thought to be resistant to radiotherapy and chemotherapy [7, 8, 10], clinical experience with our case suggests that multimodality therapy including platinum based chemotherapy is beneficial. Multi-institution studies are needed to establish a better therapeutic protocol for these rare cases.

In conclusion, this case report illustrates atypical presentation of bladder cancer in a pancreas and kidney transplant recipient. Our experience should alert physicians and radiologists of the possibility of malignancy in the differential diagnosis and the need for early biopsy to avoid diagnostic confusion with graft rejection.

## References

[1] Zani D, Simeone C, Antonelli A, Bettini E, Moroni A, Cosciani Cunico S (2008). Cancer in kidney transplantation. Urol Int.

[2] Vajdic CM, McDonald SP, McCredie MR, van Leeuwen MT, Stewart JH, Law M, Chapman JR (2006). Cancer incidence before and after kidney transplantation. JAMA.

[3] Lemmers MJ, Barry JM (1990). De novo carcinoma of the lower urinary tract in renal allograft recipients. J Urol.

[4] Gifford RR, Wofford JE, Edwards WG (1998). Carcinoma of the bladder in renal transplant patients. A case report and collective review of cases. Clin Transplant.

[5] Geetha D, Tong BC, Racusen L, Markowitz JS, Westra WH (2002). Bladder carcinoma in a transplant recipient: evidence to implicate the BK human polyomavirus as a causal transforming agent. Transplantation.

[6] World Health Organization Classification of Tumors, Infiltrating urothelial carcinoma. In: J.N. Eble, G. Sauter and J.I. Epstein et al., Editors, Tumors of the Urinary System and Male Genital Organs., IARC Press, Lyon 2004, pp. 93–110.

[7] Holmang S, Johansson SL (2001). The nested variant of transitional cell carcinoma--a rare neoplasm with poor prognosis. Scand J Urol Nephrol.

[8] Liedberg F, Chebil G, Davidsson T, Gadaleanu V, Grabe M, Mansson W (2003). The nested variant of urothelial carcinoma: a rare but important bladder neoplasm with aggressive behavior. Three case reports and a review of the literature. Urol Oncol.

[9] Volmar KE, Chan TY, De Marzo AM, Epstein JI (2003). Florid von Brunn nests mimicking urothelial carcinoma: a morphologic and immunohistochemical comparison to the nested variant of urothelial carcinoma. Am J Surg Pathol.

[10] Lin O, Cardillo M, Dalbagni G, Linkov I, Hutchinson B, Reuter VE (2003). Nested variant of urothelial carcinoma: a clinicopathologic and immunohistochemical study of 12 cases. Mod Pathol.

[11] Drew PA, Furman J, Civantos F, Murphy WM (1996). The nested variant of transitional cell carcinoma: an aggressive neoplasm with innocuous histology. Mod Pathol.

[12] Wasco MJ, Daignault S, Bradley D, Shah RB (2010). Nested variant of urothelial carcinoma: a clinicopathologic and immunohistochemical study of 30 pure and mixed cases. Hum Pathol.

[13] Sternberg CN, Yagoda A, Scher HI, Watson RC, Geller N, Herr HW, Morse MJ (1989). Methotrexate, vinblastine, doxorubicin, and cisplatin for advanced transitional cell carcinoma of the urothelium. Efficacy and patterns of response and relapse. Cancer.

[14] von der Maase H, Sengelov L, Roberts JT, Ricci S, Dogliotti L, Oliver T, Moore MJ (2005). Long-term survival results of a randomized trial comparing gemcitabine plus cisplatin, with methotrexate, vinblastine, doxorubicin, plus cisplatin in patients with bladder cancer. J Clin Oncol.

